# Perioperative Team-Based Morbidity and Mortality Conferences: A Systematic Review of the Literature

**DOI:** 10.1097/AS9.0000000000000321

**Published:** 2023-08-23

**Authors:** Aubrey Samost-Williams, Roni Rosen, Alexander Hannenberg, Melis Lydston, Garrett M. Nash, Mary Brindle

**Affiliations:** From the * Department of Anesthesia, Critical Care, and Pain Medicine, University of Texas Health Science Center at Houston, Houston, TX; † Ariadne Labs, Harvard T. H. Chan School of Public Health, Boston, MA; ‡ Department of Surgery, Memorial Sloan Kettering Cancer Center, New York City, NY; § Treadwell Library, Massachusetts General Hospital, Boston, MA; ∥ Department of Surgery, University of Calgary, Calgary, Alberta, Canada

**Keywords:** adverse event, morbidity and mortality conference, patient safety, perioperative, quality improvement

## Abstract

**Objective:**

This systematic review aimed to identify key elements of perioperative team-based morbidity and mortality conferences (TBMMs) and their impact on patient safety, education, and quality improvement outcomes.

**Background:**

Patient safety in the perioperative period is influenced by system, team, and individual behaviors. However, despite this recognition, single-discipline morbidity and mortality conferences remain a mainstay of educational and quality improvement efforts.

**Methods:**

A structured search was conducted in MEDLINE Complete, Embase, Web of Science, ClinicalTrials.gov, Cochrane CENTRAL, and ProQuest Dissertations and Theses Global in July 2022. Search results were screened, and the articles meeting inclusion criteria were abstracted.

**Results:**

Seven studies were identified. Key TBMM elements were identified, including activities done before the conference—case selection and case investigation; during the conference—standardized presentation formats and formal moderators; and after the conference—follow-up emails and quality improvement projects. The impacts of TBMMs on educational, safety, and quality improvement outcomes were heterogeneous, and no meta-analysis could be conducted; however, improvement was typically shown in each of these domains where comparisons were made.

**Conclusions:**

Recommendations for key TBMM elements can be drawn from the reports of successful perioperative TBMMs. Possible benefits of structured TBMMs over single-discipline conferences were identified for further exploration, including opportunities for rich educational contributions for trainees, improved patient safety, and the potential for system-wide quality improvement. Design and implementation of TBMM should address meticulous preparation of cases, standardized presentation format, and effective facilitation to increase the likelihood of realizing the potential benefits.

## Abstract 2

**Mini abstract:** This systematic review of perioperative team-based morbidity and mortality conferences showed that they offer potential advantages to educational and quality improvement outcomes over traditional morbidity and mortality conferences, but the robust assessment of these outcomes remains an area of needed research. Recommendations are made to aid in the implementation of team-based conferences.

## INTRODUCTION

Perioperative morbidity and mortality conferences have become nearly ubiquitous in academic medical centers since the Accreditation Council for Graduate Medical Education began requiring, in 1983, that all residency programs have a case review process in place,^1^ and are widely used in nonacademic settings as well. However, there is little standardization and high variability in conference aims, participants, case selection, and case analysis.^2^ Despite the understanding that healthcare is delivered by a team working in a system, relatively few institutions have established team-based morbidity and mortality conferences (TBMMs)^3–7^ for the perioperative setting, where TBMMs are defined by the inclusion of multiple role groups, including physicians of different specialties, such as surgeons and anesthesiologists, and clinicians of different professions, such as nurses and physicians. The morbidity and mortality conferences should also be distinguished from adverse event reviews or sentinel event reviews, which involve a small, and frequently multidisciplinary, team that investigates the cause of an adverse event, whereas a morbidity and mortality conference is designed to reach a larger group of learners and discussants to understand an incident.

The perioperative space is characterized by high acuity, with the potential for the patient’s condition to deteriorate in seconds. Multiple physicians work shoulder to shoulder around a patient, and the care they all provide can be highly interdependent, as in airway surgeries, for example, where the work of the surgeon and the management of ventilation by the anesthesiologist are tightly intertwined. Perioperative teams also include nurses and surgical technologists, who provide essential support to the surgeons and anesthesiologists. These teams often spend hours working together in close proximity in a high-stakes environment. This intensive teamwork makes perioperative TBMMs both vital and challenging, as the team dynamics from this high-stakes environment can spill into TBMMs, including the tensions between role groups and their traditional hierarchies.

To better understand how we might support the implementation of perioperative TBMMs, our systematic review was aimed at identifying factors associated with the successful implementation of perioperative TBMMs. We were also interested in assessing the strength of the evidence associating TBMMs with improvements in patient safety, quality of clinical care, and educational outcomes.

## METHODS

This review was conducted in accordance with the Preferred Reporting Items for Systematic Reviews and Meta-Analyses guidelines,^8^ and the review protocol was prospectively registered with PROSPERO, the International Prospective Register of Systematic Reviews (protocol CRD42022350149).

Electronic searches for published literature were conducted by a medical librarian using the following databases: MEDLINE Complete via EBSCO (1857 to present), Embase (1947 to present), Web of Science (1900 to present), Cochrane Central Register of Controlled Trials via Ovid (1991 to present), ProQuest Dissertations & Theses Global (1990 to present), and ClinicalTrials.gov (1999 to present). The searches were conducted in July 2022.

The search strategy incorporated controlled vocabulary and free-text synonyms for the concepts perioperative, morbidity, mortality, and conferences. The full search strategies are listed in Supplemental Material 1, http://links.lww.com/AOSO/A244. No restrictions on language or any other search filters were applied. All identified studies were combined and deduplicated in EndNote X9. The citations were then uploaded into the Covidence software.

Citations with abstracts were independently screened by two authors (A.S.W. and R.R.) for inclusion criteria. The full text was then screened in all abstracts that did not clearly fail to meet the inclusion criteria. Discrepancies in recommendations for inclusion or exclusion were resolved in consultation with a third author (M.B.).

The morbidity and mortality conference in each study included in the systematic review had to be perioperative and include personnel from more than one medical specialty (eg, anesthesia and surgery) or more than one profession (eg, physician and nurse), and in this respect, it differed from typical departmental or division case review conferences. Perioperative space was defined as any environment where a proceduralist physician might interact with a patient under the care of an anesthesia team, such as the operating room, the labor and delivery ward, the endoscopy suite, or the interventional radiology suite. Studies that were not available in English, were available only as an abstract or book chapter, or were not peer-reviewed were excluded. Review articles or perspective and opinion pieces were excluded. No study was excluded on the basis of study design.

Study quality and risk of bias were assessed using the Newcastle-Ottawa Quality Assessment Scale for Cohort Studies,^9^ with scoring done by one author (A.S.W.), followed by a discussion with the research team to reach a consensus score. Data were extracted by two authors (A.S.W. and R.R.) and checked for accuracy by the research team using a template that is available on request from the authors. Specifically, data were extracted around the hospital characteristics (academic *vs* community hospital, geographic location), the patient population addressed by the TBMMs, the characteristics of the TBMMs (actions taken before, during, and after the conference), implementation process measures (number of conferences held and attendance at conferences), and outcome measures (educational outcomes, adverse event investigation findings, and quality improvement outcomes). It was anticipated that the availability of quantitative measures of patient safety and clinician education would be limited, and no meta-analysis was planned.

## RESULTS

### Studies

**FIGURE 1.**
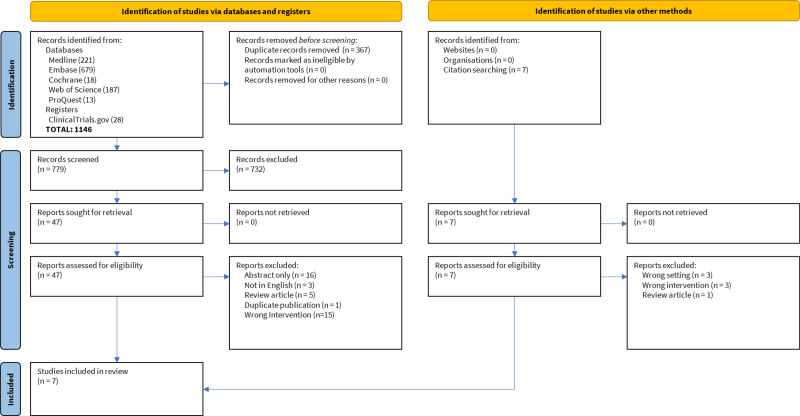
Inclusion and exclusion of studies, based on PRISMA guidelines. from: Page MJ, McKenzie JE, Bossuyt PM, Boutron I, Hoffmann TC, Mulrow CD, et al. The PRISMA 2020 statement: an updated guideline for reporting systematic reviews. BMJ 2001;372:n71. doi: 10.1136/bmj.n71. For more information, visit: http://www.prisma-statement.org/.

The database search identified 779 studies; 47 were selected for full-text review, and 7 qualified for inclusion (**Figure 1** and **Table 1**).^10–16^ One study was a national survey of pediatric surgeons. The other 6 studies typically used mixed methodologies combining surveys of conference participants, retrospective analysis of cases or interventions, and qualitative assessments of the participants’ perspectives. The studies had a moderate risk of bias, with scores of 4 or 6 stars on the Newcastle-Ottawa Quality Assessment Scale, where a score of 9 stars corresponds to the highest study quality (Supplemental Material 2, http://links.lww.com/AOSO/A244). None of the studies included controls for comparing patient groups or quality improvement projects.

**TABLE 1.** Studies Included in the Review

Study	Study Design	Sample Size	Clinical Site	Patient Population
Risucci 2003^10^	Pre-post	76 cases, 860 surveys (28 general surgery residents, 4 critical care fellows, 20 general surgery attendings, 6 medical students)	Academic medical center in the northeastern United States	Adult general surgical patients
Kauffmann 2011^11^	Retrospective	11 cases	Academic medical center in the southern United States	Adult general surgical patients
Larrazet 2011^12^	Retrospective	99 meetings, 146 cases	Nonacademic medical center in Paris, France	Adult cardiac surgical patients
Stanford 2012^13^	Pre-post	1294 CABG cases: 689 before TBMM implementation, 605 after	Nonacademic medical center in the northern United States	Adult cardiac surgical patients
Berman 2019^14^	National survey	353 survey respondents	Academic and nonacademic hospitals across the United States	Pediatric general surgical patients
Ervin 2021^15^	Mixed methods	140 survey respondents, 12 interviewees	Academic medical center in the northern United States	Adult general surgical patients
Lahnaoui 2022^16^	Retrospective	10 cases	Academic medical center in Morocco	Adult surgical oncology patients

CABG indicates coronary artery bypass graft surgery; TBMM, team-based morbidity and mortality conferences.

### Survey of Pediatric Surgeons

The survey study conducted by Berman et al^14^ examined the perspectives of 353 pediatric surgeons on the effectiveness of morbidity and mortality conferences. While not a report of a single institution’s experience like the other literature included, it provides useful, aggregated insights from a specific surgical specialty. Conferences that included participants from other departments in addition to the department of surgery were more likely to be perceived as effective (55% vs 47%; *P* = 0.005). On open-ended questions, respondents noted that the inclusion of specialists from multiple disciplines in the conference increases accountability, provides clear communication channels, and aids in the identification of complex system factors that contributed to an adverse event. The survey also identified several factors associated with conference effectiveness and engagement: feedback pathways, discussion of nontechnical components of care, standardization of case assessment and decision-making, and connection to quality improvement projects and higher-level hospital review processes. The study’s limitations include the relatively low response rate of 38% (a total of 928 pediatric surgeons were invited to complete the survey).

### TBMM Characteristics

**FIGURE 2.**
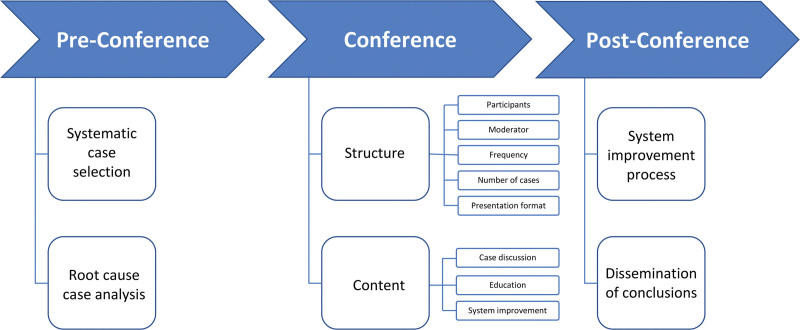
Elements of the TBMM process.

Elements of the TBMM process addressed in these studies include preparation for the meeting, the meeting itself, and postmeeting follow-up (**Fig.2**). The specific elements of the processes implemented by the TBMMs described in the studies are listed in **Table 2**.

**TABLE 2.** Features of the TBMM Process Identified in the Studies

Study	Preparation		TBMM Meeting					Follow-Up	
	Case Selection	Case Analysis	Participants	Format	Moderator	Presenter	Frequency, length	Implementation of Recommendations	Dissemination of findings
Risucci 2003^10^			Surgery trainees and faculty, interventional radiologists, physician assistants, nurse practitioners, OR and ICU nurses	Clinical summary and literature review; focus on timeliness and appropriateness of diagnoses and treatments		Residents	Weekly, 90 min (3 cases)		
Kauffmann 2011^11^	Cases with high educational value are selected by risk managers weekly	Formal root cause analysis	Environmental staff, administrators, anesthesiologists. nurses, pharmacists, radiologists, surgeons	Summary of clinical course; discussion focused on interface of systems and human factors as sources of error	Formal moderator role	Clinical team	Quarterly, 90 min	Changes proposed by an experienced clinician and the intuition’s safety officer are referred to a perioperative quality improvement committee	Updates are provided at subsequent meeting
Larrazet 2011^12^			Physicians from cardiology and critical care, cardiac surgeons, nurses.	Chronological clinical summary; discussion of how to prevent similar events		Clinical team	Weekly, 1 h (1–2 cases)	Action plans are developed by a cardiologist (TBMM leader) and a research assistant	Action plans are posted on institutional intranet
Stanford 2012^13^	All adverse events: death, reoperation, renal failure, sternal infection, pulmonary failure		Cardiac surgeons; cardiologists; nurses from the OR, ICU, catheterization lab, and cardiac floor	Fix the problem, rather than assign blame	Physician project lead		Monthly	A nurse clinical coordinator ensures that recommended changes are made	
Ervin 2021^15^	Cases recommended by faculty and staff and selected by a surgeon with expertise in failure to rescue based on presence of system issues	Collaboration between clinical team and research team to identify key turning points	Surgical faculty and trainees, clinicians from other departments, health system administrators	Identify opportunities to improve at the clinician, unit, department, and hospital levels; discussion framed around cognitive biases and research-based communication strategies	Faculty member supported by the research team	Trainee involved in patient’s care, supported by the research team	Bimonthly (1–2 cases)	Specific takeaways are identified; protocols and policies are modified accordingly	
Lahnaoui 2022^16^	Surgery team selects adverse events of grade >3a on Clavien-Dindo scale	Identification of factors using the ALARM framework^17^	Physicians and nurses from surgical and anesthesia departments	Framed around an Ishikawa diagram and 5 Whys	Senior surgeon with extra training	Resident involved in patient’s care	~Bimonthly	An action plan is developed using SMART criteria	Proposed protocol changes are posted on departmental website

ALARM indicates Association of Litigation and Risk Management; ICU, intensive care unit; OR, operating room; SMART: specific, measurable, achievable, relevant, timely.

Activities occurring before a TBMM meeting typically included in-depth case preparation^15^ and structured case analysis, such as sentinel event review or root cause analysis.^11,16^ The TBMM is thus different from such well-established fact-finding activities but builds on such processes and includes a larger group of potential learners. Ervin et al^15^ found in their qualitative analysis that in-depth case preparation was important to TBMM success and that it facilitated productive discussion around system issues. Some TBMMs discussed all adverse events or deaths at their meetings,^13,16^ whereas others carefully selected cases for educational value or potential to highlight system issues.^11,15^

All TBMMs had some standardization of presentations, with a clinical case summary typically followed by a discussion focused around a key framework, such as “fix the problem, rather than assign blame”^13^ or the cognitive biases that impact healthcare delivery.^15^ Risucci et al^10^ noted that a structured format facilitated the contextualization of an adverse event within the patient’s clinical course, from preoperative decision-making to postoperative care. This broad perspective made it possible to identify a more comprehensive array of factors that may have contributed to an adverse event and to come up with more effective solutions. Lahnaoui et al^16^ pointed out the importance of standardization in allowing participants to feel more comfortable because of clear expectations and the reduced likelihood of accusations. Five studies reported that cases were presented at TBMM meetings by clinicians, including trainees.

Four studies mentioned a formal TBMM moderator role, filled by someone with training or experience in patient safety, such as a senior surgical leader or a faculty member supported by a safety expert.^11,13,15,16^ Ervin et al.^15^ found that the presence of a moderator was perceived as important for guiding and shaping discussion. However, no formal assessment was reported to evaluate the effectiveness of moderators in facilitating open discussion free from fear, eliciting quality improvement recommendations from a broad range of participants, or enhancing educational value.

Five studies described elements of postconference follow-up.^11–13,15,16^ The most common element was the identification of key takeaway points and recommendations for practice changes. Stanford et al^13^ described a TBMM process tightly tied to updating protocols and procedures, with a clinical nurse coordinator recording protocol changes agreed to in the meeting and distributing them to physicians and nurses for implementation. At some institutions, TBMM recommendations and changes were disseminated through postings on the intranet^12,16^; at others, they were distributed at subsequent TBMM meetings.^11^

The TBMMs were diverse and included surgeons, anesthesiologists, trainee physicians, nonsurgical proceduralists, medical specialists, physician assistants, nurse practitioners, and nurses from the operating room, intensive care unit, and inpatient surgical floors. Some TBMMs also included hospital leadership, quality, and safety experts, or research team members.

### TBMM Outcomes

TBMM outcomes can be grouped into 3 categories: education, identification of adverse event causes and contributing factors, and quality improvement measures (**Table 3**).

**TABLE 3.** Outcomes and Process Measures

Study	Educational Outcomes	Adverse Event Findings	Quality Improvement Process Measures
Risucci 2003^10^	Not assessed	Post- vs preimplementation of structured format: complications judged by majority of participants to be avoidable, 54% vs 23% (*P* < 0.05); complications judged likely to have preoperative causes, 26% vs 7% (*P* < 0.01); complications judged likely to have postoperative causes, 28% vs 67% (*P* < 0.01)	Not assessed
Kauffmann 2011^11^	All 6 ACGME core Competencies addressed in most case discussions	4 of 11 cases were procedure-related, 4 were process related, 1 was patient-related, 1 involved a communication error, and 1 involved a medication error	23 quality improvement initiatives developed in 21 months; 15 completed
Larrazet 2011^12^	Not assessed	Factors to which failure was attributed most frequently: preoperative strategy, 58% of cases; surgical technique, 50%; monitoring, 47%; reactivity, 43%; drug prescription 32%	Not assessed
Stanford 2012^13^	Not assessed	Not assessed	Society of Thoracic Surgeons rating of hospital increased from 1 star to 3
Ervin 2021^15^	Participants’ diagnostic and surgical skill sets expanded	Not assessed	4 projects completed
Lahnaoui 2022^16^	Not assessed	No. of cases (n = 10) with the following contributory factors: patient, 10; tasks 9; clinical personnel, 3; team 6; work environment, 3; organization, 3	9 protocol proposals, 1 educational initiative, 1 technical proposal, 1 clinical research project, 2 communication improvements

ACGME, Accreditation Council for Graduate Medical Education.

Two studies looked at educational outcomes. Ervin et al^15^ found that providing a defined framework for discussion can increase perceived educational value compared with a free-form format. TBMM members reported that participation in the TBMM increased their diagnostic and surgical skills. Kauffmann et al^11^ reported that all TBMM cases in their study addressed 4 of the 6 core competencies established by the Accreditation Council for Graduate Medical Education^1^—Practice-Based Learning and Improvement, Interpersonal and Communication Skills, Professionalism, and Systems-Based Practice*—*and that most cases addressed the remaining 2 competencies, Patient Care and Procedural Skills and Medical Knowledge.

Four studies described TBMM investigations of adverse events aimed at identifying contributing factors, which ranged from clinical skills of the individual provider to organizational culture (**Table 3**).^10–12,16^ Risucci et al^10^ reported that a structured format helped TBMM participants recognize avoidable complications, with the majority of participants categorizing complications as avoidable in 54% of the cases, compared with 23% of the cases before the structured format was implemented.^10^ The greater recognition of avoidable adverse events led to system improvements aimed at prevention.

Three studies described TBMM-associated quality improvement initiatives (**Table 3**).^11,15,16^ Stanford et al^13^ reported that the establishment of monthly TBMMs as part of a quality improvement project helped their community hospital raise its rating from 1 star to 3 stars by the Society of Thoracic Surgery, which they attributed to improved adherence to evidence-based best practices in cardiac surgery.

## DISCUSSION

Despite the limited literature available, there are still potential best practices suggested by these studies that could aid the implementation of perioperative TBMMs. Before the conference, multiple studies discussed taking structured approaches to event analysis, which allowed for a more detailed and robust discussion at the conference. Which structured approach was utilized varied with the institution, but having a robust and structured investigation led to a perceived richer discussion. In the conference itself, standardization of TBMM presentations and procedures was reported to facilitate the identification of a more comprehensive array of factors that may have contributed to an adverse event, as well as ensure that conferences met their educational goals while creating a psychologically safe space for discussion. Conference organizers used several tactics to create this standardization of presentations, such as presentation slide templates, consistent speakers or moderators, presentation preparation assistance by quality and safety experts, and explicit statements that conferences are not for assigning individual blame. After the conference, presenting clear action items with documented follow-up allowed participants to feel that the discussion accomplished a goal and improved patient care and system functioning. The successful conferences tended to close the loop with participants on these follow-up items either at the beginning of the next conference or by email or health system intranet messages.

The data from the studies examined in this review provide evidence of the potential benefits of perioperative TBMMs and can be used to help guide institutions in implementing their own perioperative TBMMs. The reported benefits of TBMMs include recognition of avoidable adverse events, quality improvements associated with adherence to evidence-based best practices, and promotion of core competencies established by the Accreditation Council for Graduate Medical Education.

TBMMs appear to have many theoretical advantages over nonmultidisciplinary morbidity and mortality conferences. Although no robust evidence of these advantages has been obtained yet (and keeping in mind the role of publication bias in making unsuccessful TBMMs less likely to be reported in the literature), the indirect evidence that is available suggests that the inclusion of multiple disciplines contributed to the success of the TBMMs by facilitating accountability, identification of solutions to complex problems, dissemination of feedback to all perioperative staff, and follow-up on action items. The conclusions in this review are limited by the fact that the 6 studies describing TBMM implementation were all single-center studies, either describing programs or using unadjusted preimplementation and postimplementation trial designs. There remains significant space for research into the outcomes that can be impacted by TBMMs, in terms of patient care outcomes and educational outcomes. Future work can be done to understand barriers and facilitators to broader implementation of perioperative TBMMs and testing implementation science strategies and tools to leverage the facilitators and overcome the barriers. There is additionally a large space for work in understanding the impact of TBMMs on clinician education, both as trainees and as fully qualified clinicians, and patient safety metrics, which are the ultimate goals of any morbidity and mortality conference.

Another limitation of this meta-analysis is that the studies were all identified in the traditional medical literature. While the most academic analyses of the implementation of TBMMs will be found in these databases of peer-reviewed medical literature, there are likely many hospitals that have experience with TBMMs that have not published their experiences in this literature base, despite having useful lessons to share. Future work could be done in querying the gray literature on this topic—searching for blog posts, videos, or program websites—or in surveys of perioperative clinicians to understand their experiences with TBMMs that are not captured in these 6 studies.

While this review focused on the perioperative space, which has unique features in healthcare, there is a body of literature around TBMMs in other areas of medicine, for example, those described in reports from internal medicine,^3^ pediatric intensive care,^6^ and emergency medicine,^7^ among others. The studies in these diverse contexts implemented clear structures of activities before, during, and after the conference. While the exact details differ between studies and contexts, they generally identify similar characteristics of successful conferences, including formal guidelines for event identification and analysis, standardization of presentations with trained moderators, and clear follow-up items, including integration with other departmental and hospital quality improvement groups. These TBMMs were associated with increased numbers of successful quality improvement projects,^6^ perceived improvement in clinical care,^7^ and improved educational outcomes.^3^ Institutions seeking to design and implement perioperative TBMMs should draw on the literature around TBMMs in other healthcare spaces in addition to the literature from the perioperative space reviewed above.

## CONCLUSION

Clinical work in the perioperative space involves tight teamwork among surgeons, anesthesiologists, operating room nurses, and others. Discussion of adverse events in this space would benefit from the introduction of TBMMs, which have the potential to improve education for trainees and broaden the range of ideas identified that could improve the perioperative system and prevent future similar accidents. The successful TBMMs described here used structured formats for activities before, during, and after the conference to ensure that adverse events were fully investigated, discussions were team-based, action items were generated, and follow-up was ensured. The characteristics of TBMMs identified in this review and the promising findings of the studies suggest possible elements to be incorporated in TBMM design and areas for evaluation by health systems aiming to improve team communication and perioperative safety.

## ACKNOWLEDGMENT

The authors are grateful to Arthur Gelmis for editing the article.

## Supplementary Material


