# Supplementary material for: Perioperative Team-Based Morbidity and Mortality Conferences: A Systematic Review of the Literature
Source: Ann Surg Open. 2023 Aug 23;4(3):e321. doi: 10.1097/AS9.0000000000000321 (PMC10513145; doi:10.1097/AS9.0000000000000321)
Supplement: Supplementary file 1 [file as9-4-e321-s001.pdf]

Supplemental Digital Content 1. Search Strategy

MEDLINE (searched on July 25, 2022)

#1	(TI perioperative OR AB perioperative) OR (TI peri operative OR AB peri operative) OR (MH "Perioperative Medicine+") OR (MH "Perioperative Care+") OR (MH "Perioperative Period+") OR (TI intraoperative OR AB intraoperative) OR (TI preoperative OR AB preoperative) OR (TI postoperative OR AB postoperative) OR (TI intra operative OR AB intra operative) OR (TI pre operative OR AB pre operative) OR (TI post operative OR AB post operative)
#2	(MH "Congresses as Topic+") OR ((TI conference? OR AB conference?) OR (TI convention? OR AB convention?) OR (TI grand round? OR AB grand round?)
#3	(TI mortalit* OR AB mortalit*) OR (MH "Mortality+")
#4	(MH "Morbidity+") OR (TI morbidit* OR AB morbidit*)
#5	S2 AND S3 AND S4
#6	(TI "M&M" OR AB "M&M) OR (TI "M&Ms" OR AB "M&Ms") OR (TI "M&MC" AB "M&MC") OR (TI "M&MCs" OR AB "M&MCs") OR (TI morbidity and mortality meeting OR AB morbidity and mortality meeting) OR (TI morbidity and mortality meetings OR AB morbidity and mortality meetings) OR (TI "morbidity & mortality meeting" OR AB "morbidity & mortality meeting") OR (TI "morbidity & mortality meetings" OR AB "morbidity & mortality meetings") OR (TI quality improvement conference? OR AB quality improvement conference?) OR (TI risk management conference? OR AB risk management conference?)
#7	S5 OR S6
#8	S1 AND S7

Embase.com (searched on July 25, 2022)

#1	(perioperative OR 'peri operative' OR intraoperative OR preoperative OR postoperative OR 'intra operative' OR 'pre operative' OR 'post operative'):ab,ti,kw OR 'Perioperative Medicine'/exp OR 'Perioperative Period'/exp OR 'Preoperative Period'/exp OR 'Postoperative period'/exp OR 'Intraoperative period'/exp
#2	'Conference'/exp OR (conference$ OR convention$ OR 'grand round$'):ab,ti,kw
#3	mortalit*:ti,ab,kw OR 'Mortality'/exp
#4	morbidit*:ti,ab,kw OR 'Morbidity'/exp
#5	#2 AND #3 AND #4

#6	('M&M' OR 'M&Ms' OR 'M&MC' OR 'M&MCs' OR 'morbidity and mortality meeting' OR 'morbidity and mortality meetings' OR 'morbidity & mortality meeting' OR 'morbidity & mortality meetings' OR 'quality improvement conference$' OR 'risk management conference$'):ab,ti,kw
#7	#5 OR #6
#8	#7 AND #1

Web of Science (searched on July 25, 2022)

#1	TS=(perioperative OR "peri operative" OR intraoperative OR preoperative OR postoperative OR "intra operative" OR "pre operative" OR "post operative")
#2	TS=(conference? OR convention? OR "grand round?")
#3	TS=(mortalit*)
#4	TS=(morbidit*)
#5	#2 AND #3 AND #4
#6	TS=("M&M" OR "M&Ms" OR "M&MC" OR "M&MCs" OR "morbidity and mortality meeting" OR "morbidity and mortality meetings" OR "morbidity & mortality meeting" OR "morbidity & mortality meetings" OR "quality improvement conference?" OR "risk management conference?")
#7	#5 OR #6
#8	#1 AND #7

ClinicalTrials.gov (searched on July 25, 2022)

Condition or disease:	perioperative OR peri operative OR intraoperative OR preoperative OR postoperative OR intra operative OR pre operative OR post operative
Other terms:	morbidity mortality
Intervention/treatment:	Conference OR conferences OR grand round OR grand rounds OR convention OR conventions

ProQuest Dissertations & Theses Global (searched on July 25, 2022)

perioperative OR "peri operative" OR intraoperative OR preoperative OR postoperative OR "intra operative" OR "pre operative" OR "post operative"	in Anywhere except full text - NOFT
AND	
morbidity	in Anywhere except full text - NOFT
AND	
mortality	in Anywhere except full text - NOFT
AND	
Conference OR conferences OR "grand round" OR "grand rounds" OR convention OR conventions OR meeting OR meetings	in Anywhere except full text - NOFT

Cochrane CENTRAL (searched on July 25, 2022)

#1	(perioperative or peri operative or intraoperative or preoperative or postoperative or intra operative or pre operative or post operative).ti,ab.
#2	(mortalit* adj3 (conference$ or convention or conventions or grand round$)).ti,ab.
#3	(morbidit*(adj3 (conference$ or convention or conventions or grand round$)).ti,ab.
#4	2 or 3
#5	("M-&-M" or "M-&-Ms" or "M-&-MC" or "M-&-MCs" or "morbidity-and-mortality-meeting" OR "morbidity-and-mortality-meetings" OR "morbidity-&-mortality-meeting" OR "morbidity-&-mortality-meetings" or "quality improvement conference$" or "risk management conference$").ti,ab.
#6	4 or 5
#7	1 and 6

Supplemental Digital Content 2. Study Quality on the Newcastle-Ottawa Scale

Study	Selection	Comparability	Outcome	Total score[a]

Risucci 2003[15]	****		**	6 stars
Kauffmann 2011[12]	**		**	4 stars
Larrazet 2011[14]	**		**	4 stars
Stanford 2012[16]	****		**	6 stars
Ervin 2021[11]	**		**	4 stars
Lahnaoui 2022[13]	****		**	6 stars

[a]A score of 9 stars corresponds to highest quality.
